# Exploring the antibacterial and anti-biofilm properties of Diacerein against methicillin-resistant *Staphylococcus aureus*

**DOI:** 10.3389/fmicb.2025.1545902

**Published:** 2025-03-20

**Authors:** Yingying Sun, Yaozhou Wu, Yanbin Chang, Gaoling Sun, Xin Wang, Zhangping Lu, Keke Li, Xiaofang Liang, Qianqian Liu, Wenjie Wang, Lianhua Wei

**Affiliations:** ^1^School of Public Health, Gansu University of Chinese Medicine, Lanzhou, China; ^2^Department of Clinical Laboratory, Gansu Provincial Hospital, Lanzhou, China; ^3^First School of Clinical Medicine, Lanzhou University, Lanzhou, China

**Keywords:** Diacerein, MRSA, biofilm, RNA-Seq, ROS

## Abstract

**Background:**

Methicillin-resistant *Staphylococcus aureus* (MRSA) poses a significant clinical challenge due to its multidrug resistance. Diacerein (DIA), primarily used to treat degenerative joint diseases, has recently been found to exhibit antibacterial activity, though its specific antibacterial mechanisms remain unclear.

**Methods:**

The minimum inhibitory concentration (MIC) and minimum bactericidal concentration (MBC) of DIA, as well as in - vitro combination susceptibility testing, were determined using the broth microdilution method. Additionally, resistance induction assays, time-growth curve measurements, membrane fluidity, intracellular protein levels, and reactive oxygen species (ROS) were assessed. The inhibition and clearance of MRSA biofilms by DIA were evaluated using the crystal violet staining method, with bacterial morphology and biofilms observed via scanning electron microscopy and confocal laser scanning microscopy. Finally, transcriptome analysis was conducted to identify gene expression changes in MRSA treated with DIA, and RT-qPCR verification was performed.

**Results:**

The MIC and MBC of DIA against MRSA were 32 μg/mL and 128 μg/mL, respectively, and synergistic antibacterial effects when combined with ampicillin. DIA increased intracellular ROS levels and membrane fluidity in MRSA, decreased soluble protein synthesis, and altered bacterial morphology. Additionally, DIA significantly inhibited MRSA biofilm formation and disrupted pre - existing biofilms. Transcriptome analysis revealed 1,045 differentially expressed genes between the DIA-treated group and the control group, primarily involving pathways such as the tricarboxylic acid cycle, phosphorylation, ribosome metabolism, and nucleotide metabolism.

**Conclusion:**

In summary, DIA has antibacterial and anti-biofilm activities against MRSA and does not easily induce resistance. Its antibacterial mechanisms may involve multiple aspects, including bacterial protein synthesis, energy metabolism.

## Introduction

*Staphylococcus aureus* (SA) is widely distributed in the natural environment and is closely related to community and hospital infection issues. The emergence of Methicillin-resistant *Staphylococcus aureus* (MRSA) has exacerbated public health challenges associated with SA infections (Vadakkan et al., 2024). MRSA possesses multidrug resistance characteristics, posing a severe challenge to the clinical treatment of infections (Willis et al., 2022). Additionally, MRSA can adhere to medical devices or human tissue surfaces to form biofilms. Under biofilm conditions, resistance of MRSA to antimicrobial agents can increase by 10 to 1,000 times (Sang et al., 2024; Le and Otto, 2024). Therefore, there are fewer drugs capable of effectively treating infections caused by MRSA, and the clinical options are limited (Wu et al., 2024). The Centers for Disease Control and Prevention (CDC) in the United States has included MRSA on its list of serious threats to human health, and it has also been included on the list of high-priority pathogens for the development of new antimicrobial drugs by World Health Organization (WHO) (CDC, 2019; World Health Organization, 2024). However, the development of antimicrobial drugs faces numerous challenges, including high costs, a lack of new targets, and insufficient market incentives. Given this predicament, there is an urgent need to develop cost-effective, highly efficient, and low-toxicity drugs to combat MRSA infections.

In recent years, research on the antimicrobial activities of non-antibacterial drugs with known pharmacology and toxicology has become a hot topic (Liu et al., 2021; Wang et al., 2020). For example, it has been discovered that felodipine, a drug used to treat hypertension, can inhibit the formation of MRSA biofilms and aid in the clearance of persister (Zhang et al., 2022); Niclosamide, an anthelmintic drug, has been shown to exert potent antibacterial and anti-biofilm activity against *Corynebacterium striatum* (Folliero et al., 2022); and the antimalarial drug Mefloquine can restore the antimicrobial activity of colistin against multidrug-resistant *Enterobacteria* (Hu and Coates, 2021). Diacerein (DIA) is a novel interleukin (IL)-1 inhibitor that induces cartilage formation and is commonly used in clinical practice for the treatment of degenerative joint diseases (Wang et al., 2022). Recently, it has been indicated that DIA can inhibit the growth of *Enterococcus faecalis* and the formation of its biofilm (Fu et al., 2023); and the antibacterial activity of DIA against 76 Gram-positive cocci isolated from patients with bacterial keratitis. The results showed that the drug exhibited antibacterial activity against the majority of Gram-positive cocci, including 57 strains of *Staphylococcus* and 3 strains of *Enterococcus* (Okpala et al., 2024; Zhang et al., 2019).

Previous studies only measured the MIC of DIA against MRSA, but the specific mechanism behind it is still unclear. Therefore, the purpose of this study is to explore the mechanisms of the antibacterial and antibiofilm activities of DIA against MRSA. Firstly, using the broth microdilution method to determine the Minimum Inhibitory Concentration (MIC) and Minimal Biofilm Inhibitory Concentration (MBIC) of DIA against MRSA, and to explore the antibacterial effects when combined with other conventional antimicrobial agents. Secondly, through membrane fluidity experiments, measurement of reactive oxygen species (ROS), and observation of bacterial morphological changes using scanning electron microscopy (SEM), the study aims to further investigate the bacteriostatic effects of DIA. Finally, transcriptome sequencing analysis will be employed to identify possible pathways of DIA against MRSA, with subsequent validation through RT-qPCR. This research is expected to provide new insights and strategies for expanding the clinical applications of DIA, as well as new theoretical and experimental evidence for understanding the antibacterial mechanisms of other non-antimicrobial drugs.

## Materials and methods

### Bacterial strain selection

Clinically non-duplicate SA isolates were consecutively collected from a tertiary hospital in 2024. Exclusion criteria comprised: Strains failing quality control (indistinct band gradients on cefoxitin/oxacillin E-test strips); Mixed cultures (containing multiple bacterial morphotypes observed microscopically); Repeat isolates from the same patient within 30 days.

### Strain collection and growth conditions

Clinical isolates were collected from the Microbiology Laboratory of the Clinical Diagnostic Center at Gansu Provincial Hospital. We used Matrix-Assisted Laser Desorption/Ionization Time-of-Flight Mass Spectrometry (MALDI-TOF MS) technology to identify the isolates. By comparing the obtained mass spectra with the standard spectra in the database, we could accurately distinguish between MRSA and Methicillin-Sensitive *Staphylococcus aureus* (MSSA). The MRSA standard strain (USA300) was purchased from the American Type Culture Collection. Bacteria were inoculated into Mueller - Hinton (MH) broth and incubated at 37°C on a shaker at 200 rpm overnight. Subsequently, the bacterial suspension was diluted 1:10,000 with fresh MH broth and incubated for another 4–6 h to reach the logarithmic growth phase.

### MIC and minimum bactericidal concentration (MBC) determination

According to the guidelines of the Clinical and Laboratory Standards Institute (CLSI), the MIC and MBC of DIA against MRSA were determined using the standard broth microdilution method. Initially, the bacterial suspension in the logarithmic phase was diluted to a concentration of 2 × 10^6^ CFU/mL. Subsequently, Mueller Hinton Broth (MHB) was used to perform serial twofold dilutions of DIA, resulting in working concentrations ranging from 1 to 128 μg/mL. Each well of a 96-well polystyrene plate was then loaded with 100 μL of the prepared bacterial suspension and 100 μL of the drug solution. Positive control wells (100 μL MHB + 100 μL bacterial suspension) and negative control wells (200 μL MHB) were also established. The plate was incubated at 37°C for 16–18 h, and the absorbance at OD600nm was measured. Finally, 30 μL of resazurin was added to each well, and the plate was incubated in a 37°C incubator for 4–6 h for staining. A pink coloration of resazurin indicated bacterial growth. After determining the MIC, bacterial counting was performed. The culture from the MIC well and the two subsequent wells was streaked onto MHA agar plates using sterile cotton swabs, followed by incubation at 37°C for 24 h. The colonies on the MHA plates were counted using the viable count method. The lowest drug concentration with fewer than 5 colonies was considered the MBC, indicating that this concentration of the drug killed 99.9% of the bacteria. The experiment was repeated three times, with three replicates per experiment.

### *In vitro* combination antimicrobial susceptibility testing

Assessing the antibacterial activity of DIA combined with conventional antibacterial agent ampicillin busing the checkerboard method. The 2-fold serial dilutions of each compound within an appropriate concentration range were mixed to form an 8 × 8 matrix in a 96-well plate. Ampicillin were diluted horizontally, while DIA was diluted vertically. After incubation at 37°C for 18 h, the OD600nm absorbance of each well was measured using a spectrophotometer (RT - 6500, Shenzhen Rayto Life Science Co., Ltd., Shenzhen, China). An OD600nm value greater than 0.1 indicated bacterial growth. The Fractional Inhibitory Concentration Index (FICI) for two compounds (A and B) was calculated as follows: FICI = (MIC of A in combination / MIC of A alone) + (MIC of B in combination/MIC of B alone). The interaction between the two compounds is interpreted as follows: FICI ≤0.5, indicating “synergy”; 0.5 < FICI ≤1, “additive effect”; 1 < FICI ≤4, “no interaction”; FICI >4, “antagonism” (Liu et al., 2024). The experiment was repeated three times, with three replicates per experiment.

### Time-growth curve determination

A log-phase bacterial suspension was added to 4 mL of MHB, adjusted to a concentration of 2 × 10^6^ CFU/mL, and then treated with 50 mg/mL of DIA solution to achieve final concentrations of 1/2 MIC, MIC, 2 MIC, and 4 MIC. The mixture was incubated at 37°C with shaking at 200 rpm for 16–18 h. At 2-h intervals, 100 μL of the liquid was transferred to a 96-well plate, and the absorbance at OD600nm was measured. A time-growth curve was plotted, taking 24 h as one cycle. The experiment was repeated three times.

### Determination of induced resistance

We used increasing MIC values to reflect the resistance development of MRSA to Diacerein (Zhang et al., 2024). The standard strain USA300 was selected as the experimental subject. We used a 0.5 McFarland standard turbidity bacterial suspension (approximately 1.5 × 10^8^ CFU/mL), which is a widely accepted standard in microbiological experiments to ensure consistency and reproducibility. Subsequently, 50 μL of this standardized bacterial suspension was added to 4,950 μL of MHB medium for subsequent experimental operations. Bacteria were co-cultured with DIA at concentration gradients of 1/2 MIC, MIC, 2 MIC, 4 MIC, and 8 MIC. After 16–18 h of culture, the MIC value was determined. The process was repeated for 4 days, with the next higher concentration gradient of DIA used for co-culture. This cycle was repeated 20 times. After 20 passages, the mixture was further cultured for 4 days without DIA to stabilize the bacterial state. All MIC values were recorded. When the MIC value exceeded four times the original MIC value, it indicated that the bacteria had developed resistance. The experiment was repeated three times.

### Membrane fluidity measurement

The membrane fluidity of bacteria was measured using the Laurdan generalized polarization (GP) method (Pokorna et al., 2022). Log-phase bacterial suspension was centrifuged at 4,000 g and 4°C for 5 min to collect the bacterial pellet, which was then washed three times with PBS and resuspended to an OD600nm value of 0.5. Subsequently, the Laurdan fluorescent probe was added to the suspension to achieve a final concentration of 10 μM. After incubation at 37°C and protected from light for 10 min, the bacteria were washed four times with PBS and resuspended to an OD600nm value of 1.6. Then, DIA solution was added to the bacterial suspension to achieve final concentrations of MIC and 2 MIC, followed by shaking culture at 37°C for 1 h. Benzyl alcohol (BA) was used as a positive control, and sterile culture broth without DIA served as the blank control. Finally, the mixed buffer was aliquoted into a black 96-well microplate, 200 μL per well, and the fluorescence changes were measured using a fluorescence microplate reader (excitation wavelength: 350 nm, emission wavelengths: 460 nm and 500 nm). The membrane fluidity index was calculated using the formula Laurdan GP = (I460 - I500) / (I460 + I500). The experiment was repeated three times, with three replicates per experiment.

### Intracellular protein determination in bacteria

Log-phase bacterial suspension was washed three times with PBS and then resuspended in MHB, adjusting the bacterial concentration to 1 × 10^8^ CFU/mL. Subsequently, DIA was added to achieve final concentrations of 1/2 MIC, MIC, 2 MIC, and 4 MIC. The control group consisted of sterile culture broth without DIA. After incubation at 37°C for 4 h, 1 mL of the bacterial suspension was taken from each group, centrifuged at 4°C and 4,000 g for 10 min. The bacterial pellet was resuspended in 1 mL of PBS and subjected to ultrasonic disruption for 10 min. The Bicinchoninic Acid (BCA) Protein Assay Kit was then used according to the manufacturer’s instructions for measurement. The experiment was repeated three times, with three replicates per experiment.

### ROS measurement

This experiment aimed to determine the effect of DIA on intracellular ROS levels in USA300. Log-phase bacteria were collected by centrifugation at 4000 g and 4°C for 5 min, washed, and then resuspended in PBS to an OD600nm value of 0.5. DIA was added to the experimental group to achieve a final concentration of 2MIC. Two control groups were also included: a positive control in which 2′,7′ -Dichlorodihydro-fluorescein diacetate (DCFH-DA) was added without DIA, and a negative control in which neither DIA nor DCFH-DA was added. The suspensions were then incubated at 37°C for 2 h, 4 h, and 6 h. After incubation, DCFH-DA was added to the experimental group and the positive control to achieve a final concentration of 10 μM, while the negative control remained untreated. The bacteria were incubated at 37°C and protected from light for 15 min, washed, and resuspended in 1 mL of PBS. Finally, the fluorescence intensity of the bacterial suspensions was measured using a fluorescence microplate reader, with an excitation wavelength of 485 nm and an emission wavelength of 528 nm. The experiment was repeated three times, with three replicates per experiment.

### Determination of biofilm formation capacity in MRSA

Log-phase bacterial suspension was prepared in Tryptic Soy Broth supplemented with 0.34% Glucose (TSBG) medium and then diluted to 1 × 10^7^ CFU/mL. This suspension was pipetted into a 96-well plate using 200 μL per well. After incubation at 37°C for 24 h, the culture medium was gently aspirated, and the wells were gently washed three times with PBS. The wells were then fixed with formaldehyde at room temperature for 15 min. After aspirating the fixative, 200 μL of 1% crystal violet was added for staining for 15 min. The wells were then gently washed three times to remove excess dye. Finally, anhydrous ethanol was added to the wells, and incubated at 37°C for 30 min before measuring the absorbance at OD595nm. The OD value was measured every 12 h, with a cycle of 120 min, and the results were plotted in a curve.

### Determination of MBIC

Log-phase bacterial suspension was diluted to 1 × 10^7^ CFU/mL in TSBG medium, and then DIA was added to achieve final concentrations of 1 μg/mL, 2 μg/mL, 4 μg/mL, 8 μg/mL, 16 μg/mL, 32 μg/mL, and 64 μg/mL. Positive control wells (100 μL each of TSBG and bacterial suspension) and negative control wells (200 μL of TSBG) were also included. The samples were incubated at 37°C for 24 h. The culture medium was then gently aspirated, and the wells were gently washed three times with PBS. The wells were fixed with formaldehyde for 15 min, followed by aspiration of the fixative. Subsequently, 200 μL of 1% crystal violet was added to each well for staining for 15 min, and the wells were gently washed three times to remove excess dye. Finally, anhydrous ethanol was added to the wells, and incubated at 37°C for 30 min before measuring the absorbance at OD595nm. The results were plotted in a curve. The minimal drug concentration that resulted in a significantly reduced OD value compared to the positive control group was defined as MBIC. The experiment was repeated three times, with three replicates per experiment.

### The capacity of DIA for eliminating MRSA biofilms

To assess biofilm-clearing capability of DIA, 96-well plates were seeded with 200 μL of bacterial suspension at 10^7^ CFU/mL and incubated at 37°C for 24 h to allow complete biofilm formation within the wells. The culture medium was then gently aspirated, and the wells were washed three times with PBS. Subsequently, 200 μL of DIA solution was added to each well at concentrations of 1/2 MIC, MIC, 2 MIC, and 4 MIC. Positive control wells were filled with TSBG only. The 96-well plates were incubated at 37°C for another 24 h. Afterwards, the liquid was aspirated, and the wells were gently washed three times with PBS. Formaldehyde fixation followed for 15 min, after which the fixative was aspirated. Each well was then stained with 1% crystal violet for 15 min. Finally, anhydrous ethanol was added to the wells, and incubated at 37°C for 30 min before measuring the absorbance at OD595nm. The results were plotted in a curve. The drug concentration that resulted in the most significant reduction in OD value compared to the positive control indicated the best biofilm-clearing ability. The effectiveness threshold for biofilm clearance was set as a ≥ 50% reduction in OD value compared to the control group. The experiment was repeated three times, with three replicates per experiment.

### SEM observation

Log-phase bacterial suspension was diluted to 1 × 10^7^ CFU/mL and treated with DIA to achieve final concentrations of MIC and 2 MIC. An untreated control group was also prepared. To observe the morphological changes in MRSA after DIA treatment: the suspension was incubated at 37°C with a shaking speed of 200 rpm for 6 h, followed by centrifugation at 5,000 g for 5 min. To observe the effects of DIA on MRSA biofilm: Log-phase bacterial suspension at 10^7^ CFU/mL was added to sterile cell-slip wells in a 6-well plate, with the treatment group supplemented with 50 mg/mL DIA solution to reach a final concentration of MIC, while the control group received an equal volume of medium. Both groups were incubated at 37°C for 24 h, after which the medium was aspirated. The samples were then gently washed twice with PBS and fixed with 2.5% glutaraldehyde solution, stored at 4°C overnight. The samples underwent dehydration treatment with a series of ethanol solutions (40, 50, 60, 70, 80, 90, and 100%; each step for 10 min) before being observed under a scanning electron microscope.

### Confocal laser scanning microscopy (CLSM) observation

CLSM for observing MRSA growth status: the log-phase bacterial suspension was co-cultured with DIA at MIC concentration for 4 h, with 3 mL of SYTO 9/PI dye added to each milliliter of bacterial suspension. The mixture was thoroughly pipetted and incubated at room temperature in the dark for 10 min. Subsequently, the treated bacterial suspension was pipetted onto slides and observed and photographed under the confocal laser scanning microscope.

CLSM observation of MRSA Biofilm: firstly, MRSA bacterial suspension cultured to logarithmic growth phase was diluted to 1 × 10^7^ CFU/mL with TSBG medium. Then, in a confocal microscope-specific culture dish, MRSA was co-cultured with DIA solutions at concentrations of 1/2 MIC, MIC, and 2 MIC, respectively, and a blank control group without DIA was also set up. After incubating the culture dish at 37°C for 24 h, the culture medium was aspirated, and the dish was gently washed three times with sterile PBS. Subsequently, 500 μL of prepared SYTO 9 and PI fluorescent dyes were added to the culture dish, incubated in the dark for 15 min, and then the dye solution was aspirated. The biofilm was observed under a CLSM.

### Transcriptome sequencing

Pretreatment: Three independent samples of the drug-treated group (1/2 MIC DIA) and the blank control group of USA300 were cultured for 6 h at 37°C. Subsequently, bacterial suspensions were centrifuged at 5,000 g for 5 min to precipitate the cells. RNA Sequencing: first, the quality of the extracted total RNA was assessed to ensure its integrity, purity, and concentration, which is crucial for the success of subsequent experiments. After confirming its quality, ribosomal RNA (rRNA) was removed to enrich messenger RNA (mRNA) and other non-coding RNAs, enhancing detection efficiency. Next, long RNA chains were fragmented into smaller pieces, and double-stranded cDNA was synthesized via reverse transcription, converting RNA information into stable cDNA. Subsequently, the ends of the cDNA were repaired to make them even, and an A base was added to the 3′ end for adapter ligation. Adapted cDNA was then sorted, selecting fragments of appropriate length to ensure consistency. Afterward, double-stranded cDNA containing U was removed, and PCR amplification was performed to increase the number of target fragments. Finally, the quality of the constructed library was checked to ensure that its concentration, fragment size, and purity met sequencing requirements. Once confirmed as acceptable, high-throughput sequencing using the Illumina technology was performed, providing the foundation for subsequent data analysis.

### Transcriptome data analysis

Initial quality control of raw data involves removing reads of low quality and those containing adapter sequences. The data is then aligned with the reference genome, upon which SNV analysis and specific analyses for prokaryotes are conducted. Next, analyses of gene structure, UTRs, and sRNA annotation are performed, predicting new transcripts. Subsequent steps include the analysis of gene expression levels and strand-specific expression abundance to identify differentially expressed genes. An overall quality assessment of the RNA-Seq data is also conducted, including saturation analysis, uniformity analysis, and clustering analysis. Finally, GO enrichment analysis, COG annotation, and Pathway enrichment analysis are performed to comprehensively analyze genes from the perspectives of biological processes, cellular components, molecular functions, functional categories, and biological pathways. The criteria for defining significantly differentially expressed genes are an FDR (False Discovery Rate) < 0.05 and |log2Fold Change| ≥ 1.

### RT-qPCR validation

Total RNA was extracted from MRSA USA300 using the RNeasy Mini Kit (Qiagen, Frankfurt, Germany), following the manufacturer’s instructions. Next, 500 ng of purified RNA was reverse transcribed into cDNA using the RT Master Kit (Takara, Shiga, Japan). Following, the PCR reaction mixture was prepared using the TB Green Premix Ex Taq™ Kit (Takara, Shiga, Japan) according to the manufacturer’s instructions, and real-time PCR was performed in triplicate using the QuantStudio™ 7 Flex system (Applied Biosystems, Norwalk, USA). The amplification parameters were set as follows: an initial denaturation stage at 95°C for 30 s, followed by 40 cycles each consisting of 5 s at 95°C, 30 s at 60°C, and 45 s at 72°C. The expression of 16 s rRNA was used as a standard, and changes in other genes were analyzed by the comparative CT method. The primers used in PCR are listed in Supplementary Table S1.

### Statistical analysis

For the variance analysis in this study, one-way ANOVA was performed using GraphPad Prism 8.0.2. Group differences were considered statistically significant when *p* < 0.05 (*p < 0.05, **p < 0.01, ***p < 0.001). The statistical method of analysis of variance (ANOVA) was employed for the analysis of ROS, BCA, membrane fluidity, and the experiments related to biofilm determination, with a significance threshold of *α* = 0.05.

### Ethical statement

The collection of bacterial strains was conducted in accordance with relevant clinical guidelines. Ethical approval was not required for this study as all patient-identifiable information was removed during the experimental process.

## Results

### Analysis of the antibiotic resistance characteristics of 115 clinical isolates of SA

The antibiotic resistance characteristics of 115 clinical isolates of SA were analyzed, as shown in Table 1. Among the 115 SA isolates, there were 41 MRSA and 74 MSSA. The resistance rates of MRSA to penicillin G and oxacillin were 100%, while MRSA showed 100% sensitivity to minocycline, rifampin, daptomycin, linezolid, vancomycin, cefazoline, tigecycline, and teicoplanin. Additionally, MRSA exhibited high resistance rates to clindamycin, erythromycin, and tetracycline. MSSA showed high resistance rates to penicillin G, clindamycin, and erythromycin, but 100% sensitivity to oxacillin, minocycline, daptomycin, linezolid, vancomycin, cefazoline, tigecycline, and teicoplanin.

**Table 1:** Resistance and sensitivity rates of 115 *Staphylococcus aureus* strains.

Antimicrobial agent name	MRSA (*n* = 41)		MSSA (*n* = 74)	
	Resistance (%)	Sensitivity (%)	Resistance (%)	Sensitivity (%)
Penicillin G	100	0	90.5	9.5
Oxacillin	100	0	0	100
Co-trimoxazole	2.4	97.6	24. 3	75.7
Clindamycin	87.8	12.2	54.1	45.9
Erythromycin	90.2	9.8	56.2	43.8
Tetracycline	48.4	51.6	8.2	91.8
Minocycline	0	100	0	100
Rifampin	0	100	0	98.6
Daptomycin	0	100	0	100
Linezolid	0	100	0	100
Vancomycin	0	100	0	100
Ciprofloxacin	31	58.5	6.8	89.2
Gentamicin	12.5	87.5	11	86.3
Levofloxacin	22	75.6	6.8	93.2
Ceftobiprole	0	100	0	100
Tigecycline	0	100	0	100
Moxifloxacin	13.8	82.8	5	93.3
Teicoplanin	0	100	0	100

### Antibacterial activity of DIA against MRSA

**Figure 1:**
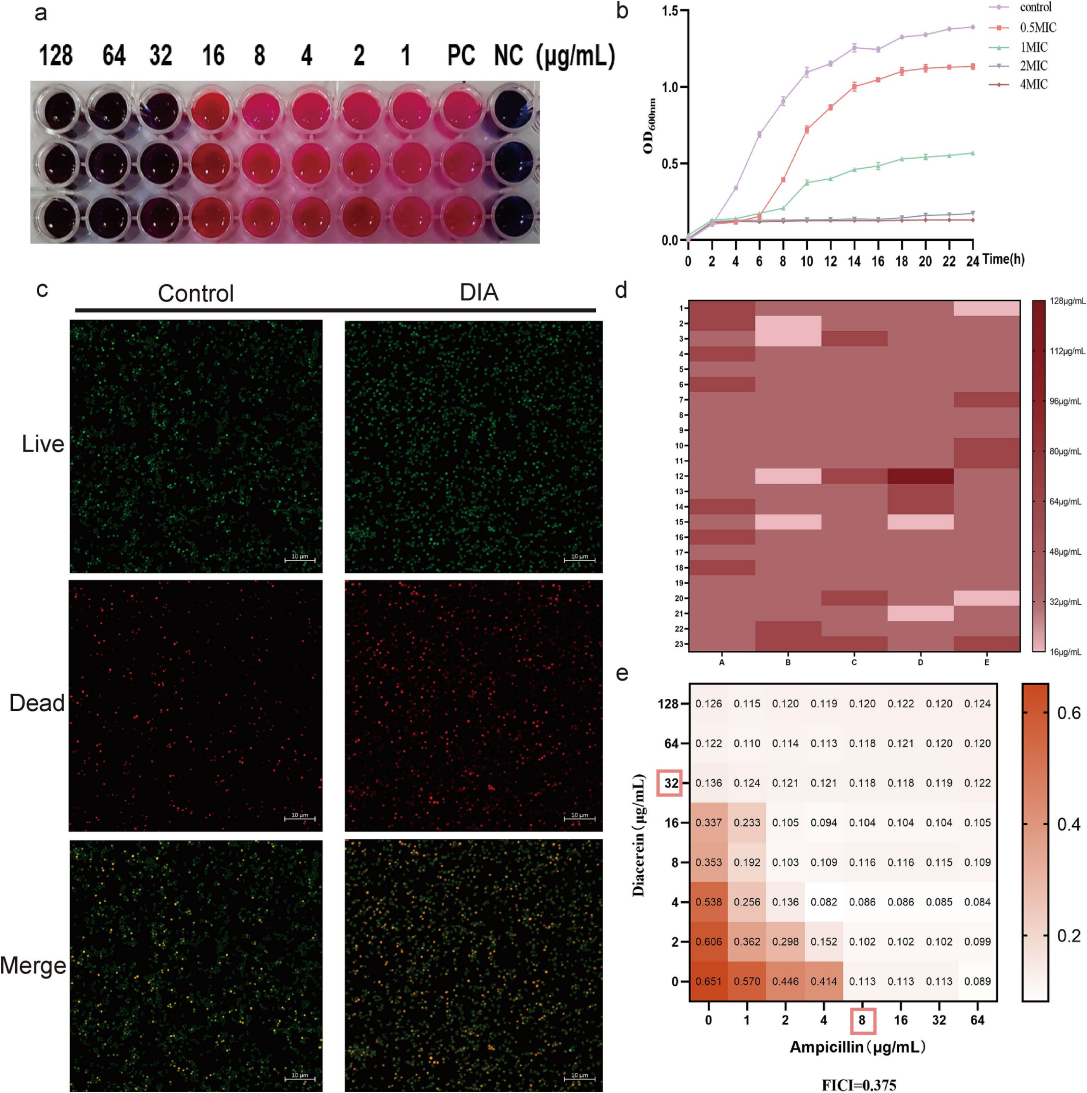
DIA exhibits antibacterial activity against MRSA. **(a)** Determination of the MIC of DIA against USA300; **(b)** Time-growth curve assessment; **(c)** CLSM observation of USA300 growth status; **(d)** Determination of MIC_50_ and MIC_90_; **(e)** Combination antimicrobial susceptibility testing of DIA with ampicillin. (NC, Negative control; PC, Positive control).

To initially verify the antibacterial activity of DIA against MRSA, we employed the broth microdilution method to determine the MIC of DIA against USA300, as shown in Figure 1a, with an MIC of 32 μg/mL. We also conducted time-growth curve experiments, and the results are shown in Figure 1b. Compared to the DIA-treated group, the blank control group entered the logarithmic phase around 4 h and reached the plateau phase after 16–18 h; after treatment with a concentration of 1/2 MIC of DIA, bacteria entered the logarithmic growth phase after 6–8 h of culture; the MIC-treated group started to proliferate in large quantities after 10–12 h of culture, indicating that bacterial growth was inhibited. The OD values of bacteria in the 2 MIC and 4 MIC DIA-treated groups barely changed. To further observe the inhibitory effect of DIA on the growth of MRSA, we used a CLSM to compare the growth status of USA300 under conditions with and without DIA, as shown in Figure 1c. Compared with the control group, after being treated with DIA at the MIC concentration, the number of dead bacteria of USA300 stained red increased significantly, and the bacteria changed from an aggregated state to a scattered distribution. Subsequently, we performed MIC determination on the 115 clinical isolates, as shown in Figure 1d. The MIC_50_ and MIC_90_ of the 115 clinical isolates were 32 μg/mL and 64 μg/mL, respectively, and the MIC_50_ and MIC_90_ of the 74 MRSA and 41 MSSA isolates were both 32 μg/mL and 64 μg/mL, respectively. At the same time, the MBC of DIA against USA300 was determined to be 128 μg/mL. Taken together, these results initially indicate that DIA possesses antibacterial activity against MRSA.

*β*-lactam antibiotics, known for their potent antibacterial capabilities, have long been the preferred choice for treating SA infections. However, MRSA exhibits resistance to these agents. To determine the combined effects of DIA with β-lactam antibiotics, we conducted a checkerboard method combined with a drug sensitivity test for screening, with the results shown in Figure 1e. When combined with ampicillin, the FICI of DIA was 0.375, indicating a synergistic antibacterial effect between DIA and ampicillin. A combination of 1/4 MIC of DIA and 1/4 MIC of ampicillin was sufficient to inhibit the growth of USA300. These results further demonstrate that DIA exhibits significant inhibitory activity against MRSA and has a synergistic antibacterial effect when combined with ampicillin.

### DIA can increase the intracellular ROS content in MRSA

**Figure 2:**
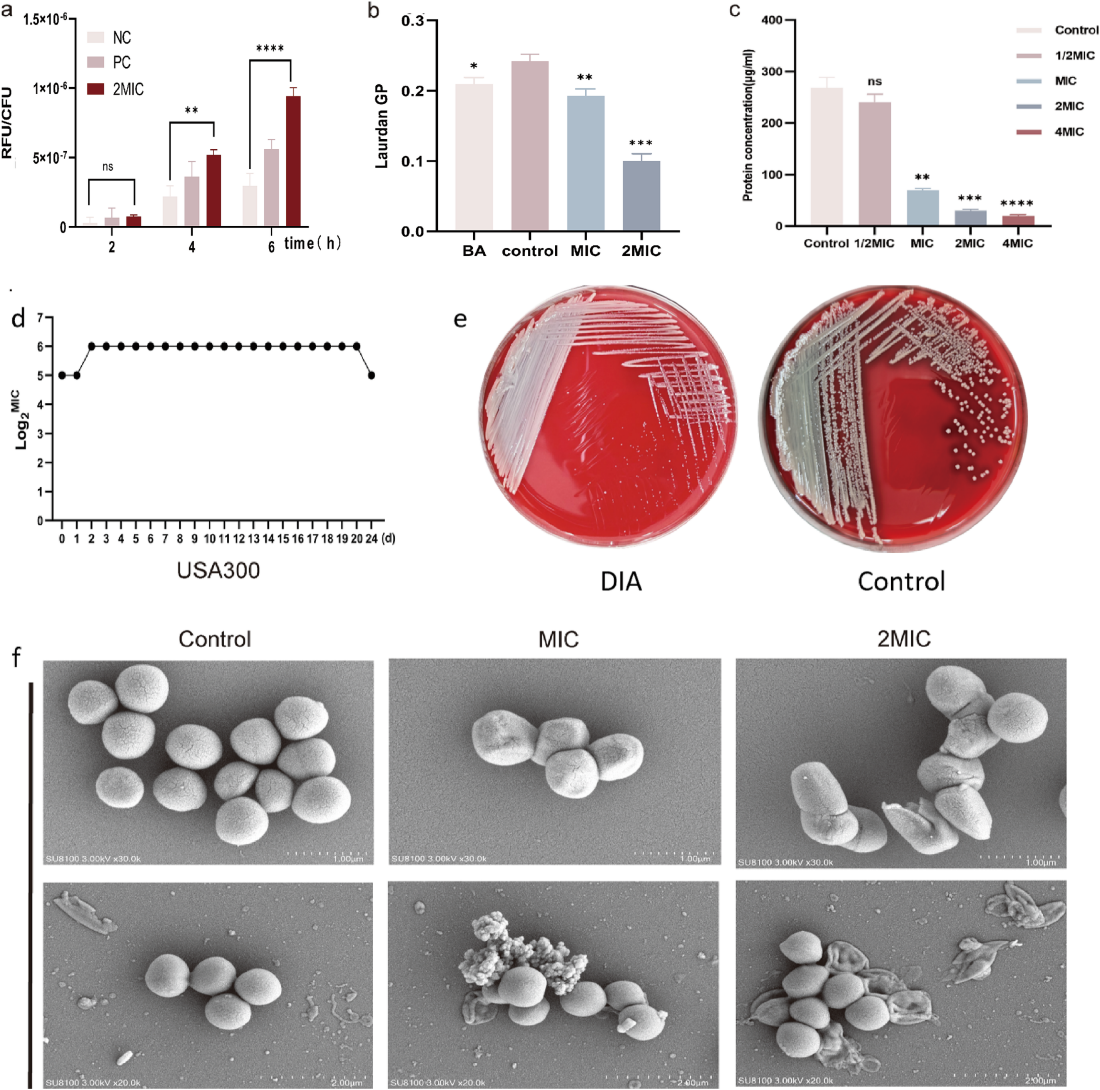
DIA increases ROS and membrane fluidity, reduces the synthesis of soluble proteins and affects the morphology of MRSA. **(a)** Determination of intracellular ROS content in MRSA; **(b)** Detection of MRSA cell membrane fluidity; **(c)** Determination of soluble protein content within bacterial cells; **(d)** Induced MIC determination for USA300 resistance; **(e)** Colony morphology changes of USA300 on Columbia blood agar plates; **(f)** SEM observation of bacterial morphology (NC: Negative control; PC: Positive control; BA: Benzyl alcohol; ns, not significant; *, *p* < 0.05; **, *p* < 0.01; ***, *p* < 0.001). The statistical method of analysis of variance (ANOVA) was employed for the analysis of ROS, BCA, and membrane fluidity.

DCFH-DA can penetrate the cell membrane and be hydrolyzed by esterase into DCFH within the cell. The non-fluorescent DCFH reacts with ROS inside the cell to produce fluorescent DCF, and the fluorescence value can reflect the intracellular ROS content of bacteria. The intracellular ROS content of USA300 was measured using the DCFH-DA fluorescent probe, and the results are shown in Figure 2a. Compared to the control group, there was no significant change in intracellular ROS content in USA300 treated with 2MIC concentration of DIA for 2 h (*p* = 0.4160, 95%CI = −15.58 ~ 6.225); however, after 4 and 6 h of treatment, the intracellular ROS content in the treated group was significantly higher than that in the control group (*p* < 0.05). This indicates that DIA can gradually increase the ROS content in MRSA over time.

### DIA can increase the membrane fluidity of MRSA cells

Laurdan GP is a standardized ratio of fluorescence emission intensities that is negatively correlated with membrane fluidity, and 50 mM BA was used as a positive control. As shown in Figure 2b, compared to the blank control group, the membrane fluidity increased in the BA-treated group (*p* = 0.0146, 95%CI = -0.05821 ~ −0.007154), and it also increased in the MIC and 2 MIC DIA-treated groups (*p* < 0.05). This indicates that DIA treatment of USA300 can increase the leakage of intracellular substances, which is beneficial for the clearance of persister bacteria.

### DIA can reduce the synthesis ability of intracellular soluble proteins in MRSA

A standard curve was generated with BCA protein concentration on the x-axis and absorbance on the y-axis, and the results are shown in the figure with a correlation coefficient R^2^ of 0.9919, indicating a strong linear relationship. Based on the measurements, the functional relationship between the OD value and protein concentration is *y* = 0.9919x + 0.1712. The concentration of BCA protein was calculated using the measured OD values, and the results are shown in Figure 2c. Compared to the control group, treating USA300 with 1/2 MIC concentration of DIA resulted in a decrease in intracellular protein content, though not significantly (*p* = 0.0798, 95%CI = −2.817 ~ 58.55). However, when treated with MIC, 2 MIC, and 4 MIC concentrations of DIA, the intracellular soluble proteins in the bacteria decreased significantly (*p* < 0.01), with the decrease becoming more pronounced as the drug concentration increased. This suggests that DIA can reduce the synthesis capacity of intracellular soluble proteins in MRSA.

### MRSA is not prone to developing resistance to DIA

Through resistance induction experiments, we explored whether MRSA would easily develop resistance to DIA. A total of 20 passages were conducted, and changes in the MIC value were continuously monitored. The MIC of USA300 only increased from 32 μg/mL to 64 μg/mL, indicating that MRSA does not easily develop resistance to DIA (Figure 2d). Notably, we observed that the colony morphology of the induced strains changed on Columbia blood agar plates, as shown in Figure 2e. Compared to the non-induced strains, the induced bacterial colonies were significantly smaller and did not exhibit a clear hemolysis ring. To rule out potential contamination during inoculation, we conducted mass spectrometry identification of their induced strains. The results showed that they all were SA, with no contamination issue (Supplementary Figure S1).

### DIA can influence the morphology of MRSA

Drugs can exert their antibacterial effects by altering bacterial morphology; some drugs can induce bacteria to transition from their normal to abnormal forms with incomplete cell walls, which can impact viability of the bacteria. Additionally, drugs can directly affect the integrity and function of the membrane, leading to holes or cracks in the bacterial cell membrane, thereby affecting its permeability and function. Using a SEM, we observed morphological changes in USA300 before and after treatment with DIA, as shown in Figure 2f. The control group bacteria were spherical, uniform in size, with regular, smooth surfaces and were intact, showing no folds or damage. After treatment with an MIC concentration of DIA for 4 h, folds and depressions began to appear on the bacterial surface. The bacteria in the 2 MIC treatment group showed obvious irregularities and even ruptures. This indicates that DIA can affect the integrity of the MRSA cell wall and cell membrane, altering its morphology and even causing direct damage.

### DIA exhibits anti-biofilm activity against MRSA

**Figure 3:**
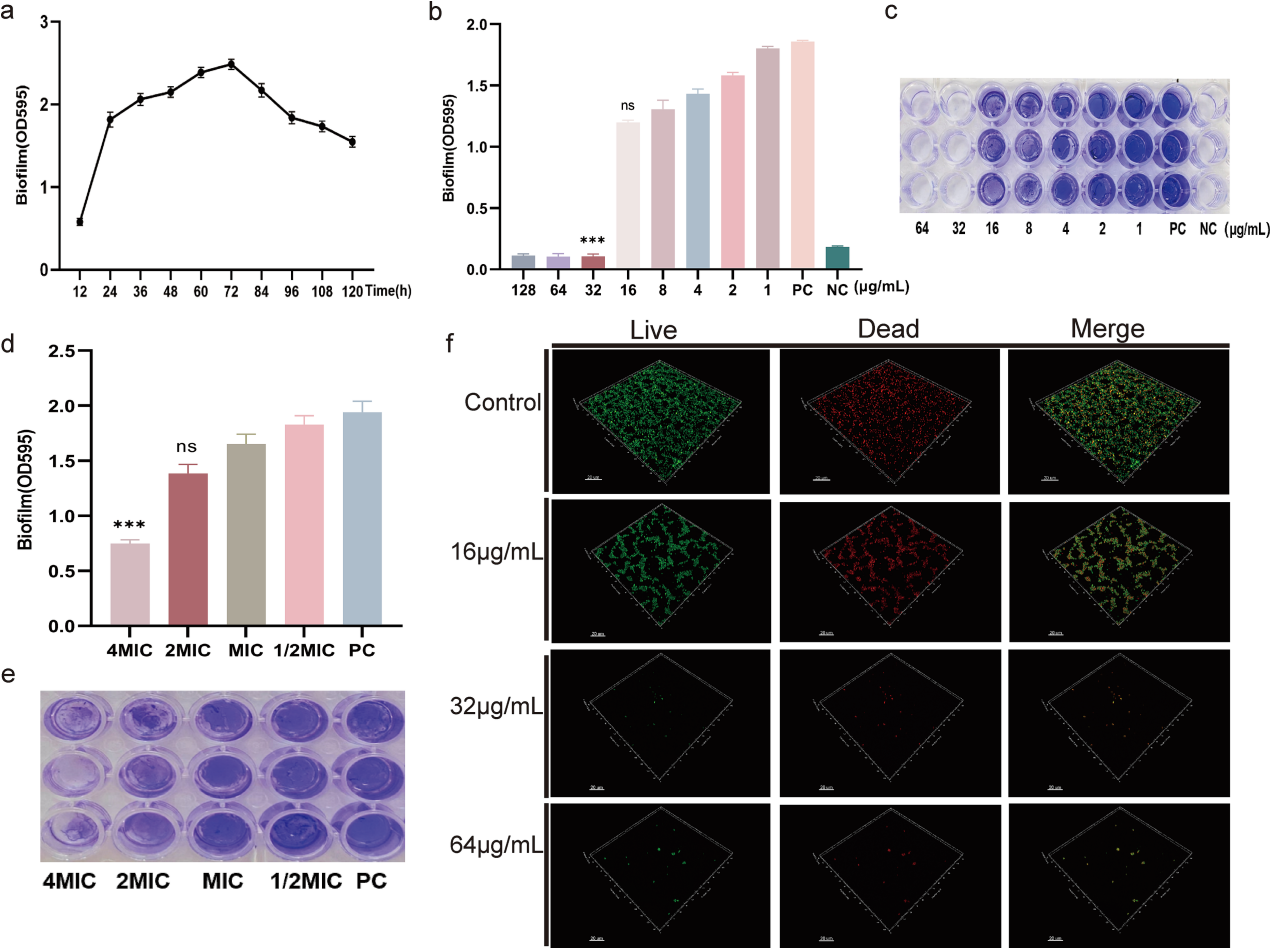
DIA exhibits anti-biofilm activity. **(a)** Formation of MRSA biofilm; **(b)** Determination of MBIC by measuring the absorbance at OD595nm; **(c)** Determination of MBIC by crystal violet staining; **(d)** Determination of the clearance effect on preformed MRSA biofilm by measuring the absorbance at OD595nm; **(e)** Determination of the clearance effect on preformed MRSA biofilm by crystal violet staining; **(f)** CLSM observation of the effect of DIA on MRSA biofilm. (NC: Negative control; PC: Positive control; ns: no significant difference; ***, *p* < 0.001).

To investigate whether DIA possesses anti-biofilm activity against MRSA, it’s first necessary to understand the formation of biofilms. We measured the biofilm-forming capability of USA300 over 120 h, with the results shown in Figure 3a. The OD value increased rapidly between 12 and 24 h and peaked between 60 and 72 h, indicating that USA300 has the strongest biofilm-forming ability during this period. Subsequently, we used the crystal violet staining method to determine the inhibitory effect of DIA on USA300 biofilms. As shown in Figures 3b,c, the positive control group without DIA had intact biofilms, while 16 μg/mL of DIA caused the USA300 biofilm to disperse. When the concentration of DIA was increased to 32 μg/mL, USA300 hardly formed any biofilms. Therefore, the MBIC of DIA against USA300 is 32 μg/mL.

To further determine whether DIA can eliminate existing biofilms, we used the crystal violet staining method to measure the clearance effect of DIA on pre-formed MRSA biofilms. The results shown in Figures 3d,e indicate that, compared to the positive control, a 4 MIC concentration of DIA has a good clearance effect on pre-formed biofilms. The 4 MIC treatment group showed a 61.41% reduction in biofilm OD value compared to the PC group (*p* < 0.0001). The 2 MIC treatment group exhibited a 28.73% reduction (*p* = 0.0085), while the MIC treatment group showed a 14.87% reduction (*p* = 0.0046), all of which were statistically significant. However, the 1/2 MIC treatment group only showed a 5.77% reduction (*p* = 0.3160).

**Figure 4:**
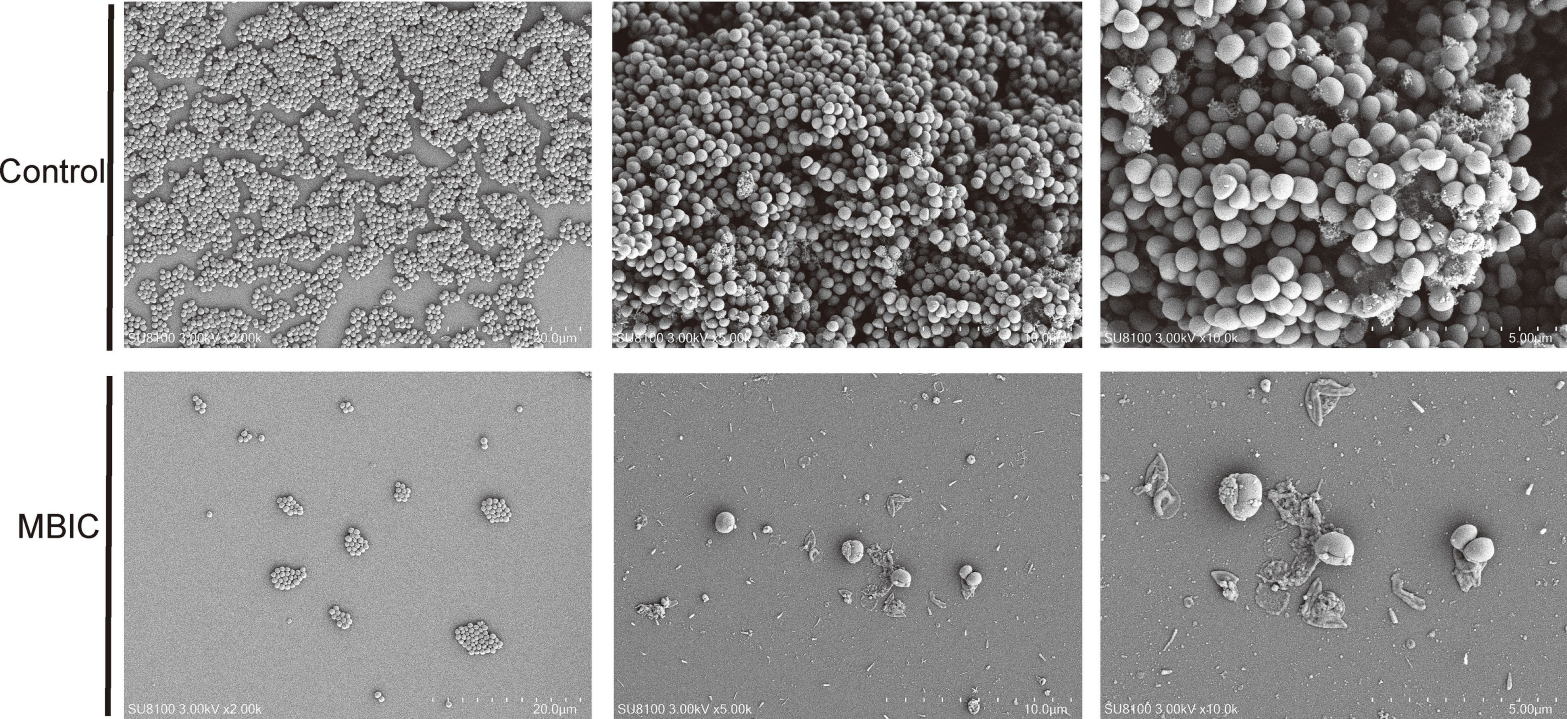
SEM observation of the inhibition of DIA on MRSA biofilm.

To more directly observe the inhibitory effect of DIA on MRSA biofilm formation, we used CLSM to observe the impact of DIA on USA300 biofilms, as shown in Figure 3f. The blank control group had a large area of green fluorescence in the 3D structure, densely distributed, forming a thick and dense biofilm structure, indicating a high number and vitality of USA300 bacteria. As the concentration of DIA increased, the area of green fluorescence in the 3D structure gradually decreased, and the structure became loose, making it difficult to form a solid biofilm structure. Using scanning electron microscopy to observe the effect of DIA on USA300 biofilms, as shown in Figure 4, the blank control showed polysaccharides forming on the bacterial surface, with bacteria stacking together to form a three-dimensional structure. After treatment with an MBIC concentration of DIA, the three-dimensional structure of the biofilm was damaged, and bacteria were scattered on the surface of the slide. These results indicate that DIA can reduce MRSA adherence, inhibiting the formation of a three-dimensional biofilm structure.

### Transcriptomic analysis

**Figure 5:**
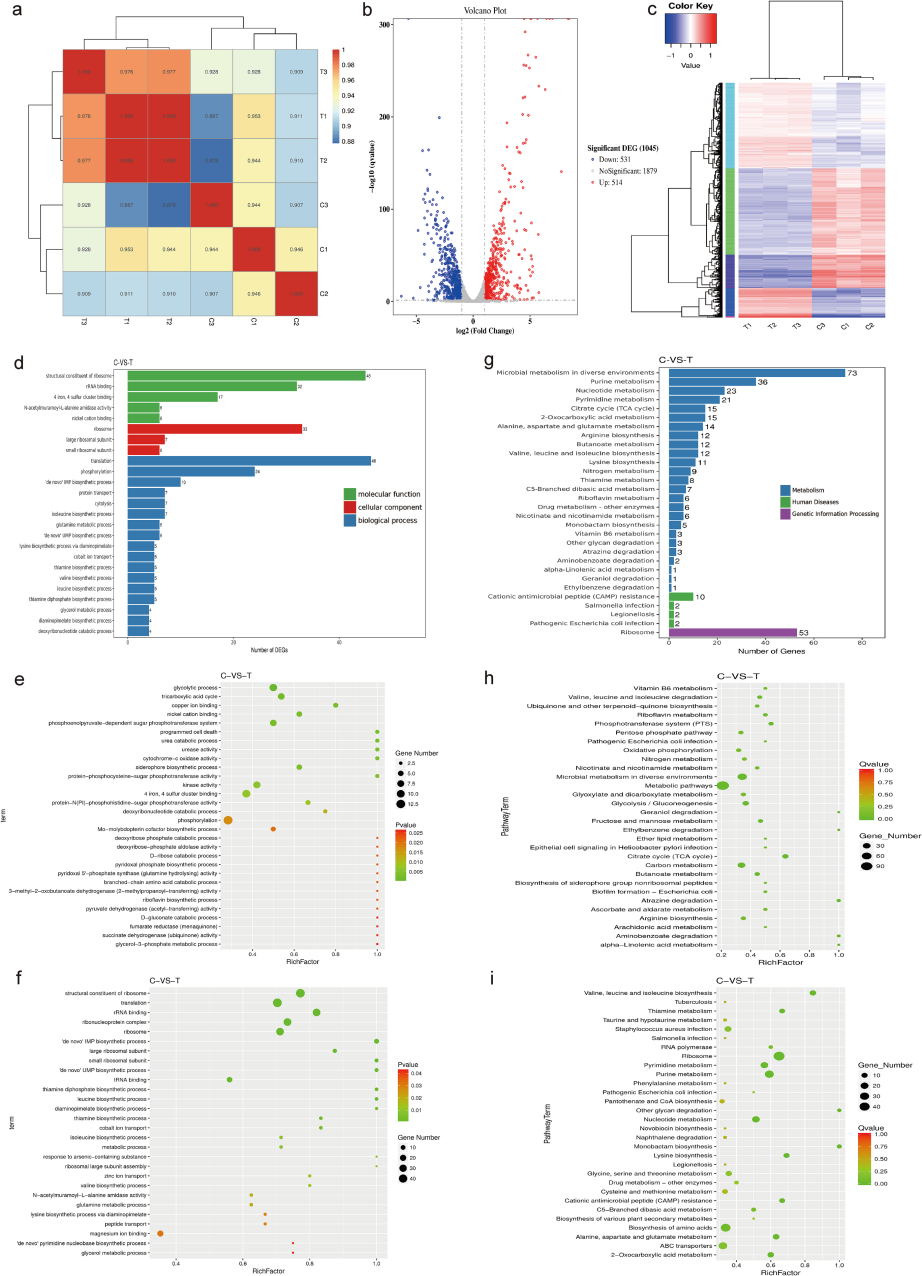
Transcriptomic analysis. **(a)** RNA-Seq correlation check; **(b)** Volcano plot of differential genes; **(c)** Clustering diagram of differential genes. Red represents highly expressed genes and blue represents lowly expressed genes; **(d)** GO enrichment bar chart of differential genes; **(e)** Scatter plot of upregulated GO distribution of differential genes; **(f)** Scatter plot of downregulated GO distribution of differential genes; **(g)** Bar chart of significantly enriched KEGG annotation categories; **(h)** Scatter plot of upregulated KEGG enrichment of differential genes; **(i)** Scatter plot of downregulated KEGG enrichment of differential genes. (C: Blank control group, T: DIA treatment group).

We used RNA sequencing to observe the transcriptional changes in MRSA bacteria after treatment with DIA, aiming to explore its antibacterial mechanisms. Our correlation analysis of RNA-Seq data showed that all Pearson correlation coefficients were greater than 0.88, indicating a high level of correlation in gene expression levels between different samples. This suggests that the selection of samples during the experiment was reasonable (Figure 5a).

In this study, after treating USA300 with a sub-inhibitory concentration of DIA, a total of 1,045 differentially expressed genes (DEGs) were identified. Among them, 514 genes were upregulated and 531 genes were downregulated. This finding indicates that DIA significantly affects the gene expression of MRSA (Figure 5b). To better understand and categorize these differentially expressed genes, genes with similar functions or close associations were clustered into different categories, and the gene expression patterns of the DIA-treated group were compared with those of the control group in these categories. The results show that the DIA-treated group differs significantly from the control group in the gene expression patterns of multiple categories, which is visually presented in Figure 5c.

After obtaining the significantly differentially expressed genes, we performed a classification and statistical analysis of Gene Ontology (GO) annotations, selecting the 25 most significantly enriched GO terms to be displayed (Figure 5d). Among them, the GO terms enriched by upregulated DEGs in the treated group were mainly related to “phosphorylation,” “glycolytic process,” and “tricarboxylic acid cycle,” while the GO terms enriched by downregulated DEGs were primarily related to “translation,” “structural constituent of ribosome,” and “ribonucleoprotein complex” (Figures 5e,f). These results reveal the biological differences and potential mechanisms between the samples, which are of great significance for subsequent research.

To determine the metabolic pathways influenced by DIA in MRSA, we analyzed the pathway annotations of genes with significant differential expression, introducing the rich factor index to measure pathway enrichment. The 30 most significantly enriched pathways (with the smallest Q value) were selected for display (Figure 5g). Seven metabolic pathways were significantly enriched, including “Microbial metabolism in diverse environments,” “Ribosome,” “Purine metabolism,” “Nucleotide metabolism,” “Pyrimidine metabolism,” “Cirate cycle (TCA cycle),” and “2-Oxocarboxylic acid metabolism.” Among these, the pathways that were upregulated mainly included “Microbial metabolism in diverse environments” and “Cirate cycle (TCA cycle).” The downregulated pathways mainly included “Biosynthesis of amino acids,” “Purine metabolism,” “Nucleotide metabolism,” “Pyrimidine metabolism,” “2-Oxocarboxylic acid metabolism,” and “Ribosome” (Figures 5h,i).

### RT-qPCR validation

**Figure 6:**
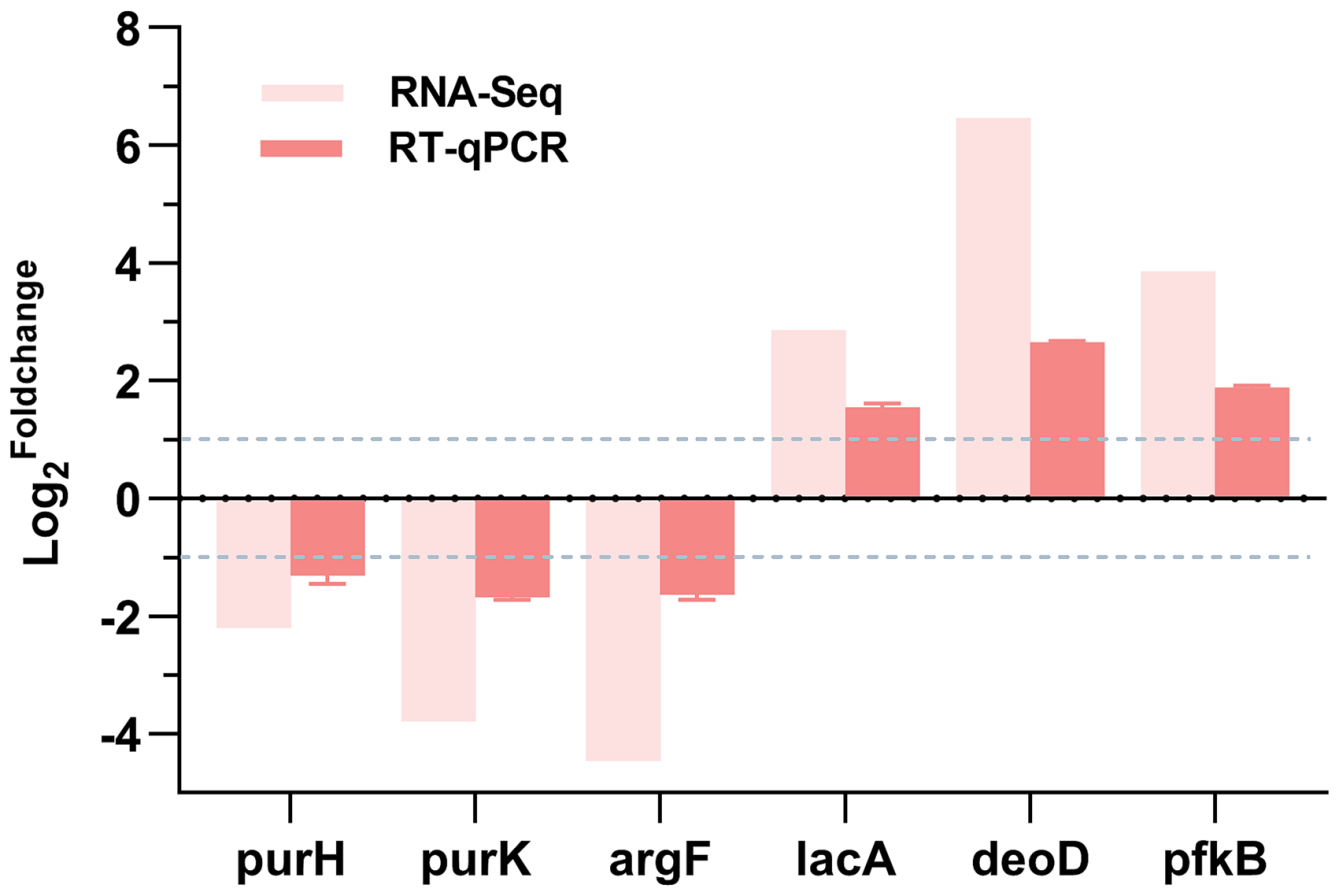
RT-qPCR validation. log^2^Foldchange: The logarithm to the base 2 of the ratio of gene expression levels between the experimental group and the control group. A positive value represents the up - regulation of gene expression in the experimental group, while a negative value represents the down - regulation of gene expression in the experimental group.

To validate the reliability of the transcriptomic sequencing results, we used RT-qPCR technology. We randomly selected 6 DEGs from the set of differentially expressed genes, namely *purH*, *purK*, *argF*, *deoD*, *pfkB*, and *lacA*, and performed RT-qPCR analysis on them. As shown in Figure 6, the trends of up-or down-regulation of the selected 6 DEGs in the DIA-treated group compared to the control group were consistent with the results from transcriptomic sequencing. This indicates that the transcriptomic sequencing results are true and reliable.

## Discussion

Through a series of phenotypic experiments and transcriptomic sequencing analysis, we investigated the antimicrobial activity and mechanisms of action of DIA against MRSA. The study found that DIA has significant antibacterial activity against MRSA and does not easily induce resistance in MRSA, exhibiting synergistic antibacterial activity when combined with ampicillin. Additionally, DIA increases MRSA membrane fluidity, promotes the release of ROS, reduces the synthesis of soluble proteins within bacterial cells, and effectively inhibits the formation of MRSA biofilms, also having a good clearing effect on existing biofilms. To further elucidate the specific antibacterial mechanisms of DIA against MRSA, we utilized transcriptomic sequencing technology to conduct in-depth analysis from gene GO enrichment and KEGG enrichment, revealing the molecular-level mechanisms of DIA, which provides key information for understanding its antibacterial mechanism.

After preliminary verification of the antibacterial activity of DIA against MRSA, to further investigate whether DIA easily induces resistance in MRSA, we conducted resistance induction experiments. Although the drug does not easily induce resistance in MRSA, compared to the non-induced strain, the induced strain after 20 passages showed significantly smaller colonies on Columbia blood agar plates, with slower growth and disappearance of the clear hemolysis zone. This indicates that while the formation of resistance is difficult, DIA may still have certain effects on the growth characteristics of MRSA. We speculate that DIA may influence bacterial cell wall and protein synthesis, as well as genes related to nucleic acid metabolism pathways, leading to mutations in these genes, thereby inhibiting bacterial growth and ultimately resulting in small colony variant (SCV) phenotypes (Becker, 2023). SCV refers to a subpopulation of bacteria that grows slowly under specific selective pressures (Zhou et al., 2022). Previous reports have indicated that SCV in SA has the characteristics of small colony size and phenotypic stability, and this is associated with its extended survival time within host cells (Guo et al., 2022). Zheng et al. (2021) detected three SCVs from 41 rifampicin-resistant SA strains. Observations on MHA blood agar plates revealed that SCVs exhibited slow growth, with colonies still being small after 32 h of incubation, reduced pigmentation, and indistinct hemolytic zones. Staudacher et al. (2024) gradually induced linezolid and tedizolid in MSSA isolates and found that tedizolid-induced resistant strains developed SCV, which was verified through whole-genome sequencing. These findings suggest that DIA may exert profound impacts on the growth and survival of MRSA by inducing the formation of SCV.

The ROS system comprises components such as superoxide, hydrogen peroxide, and hydroxyl radicals. At low concentrations, ROS can serve as signaling molecules to promote bacterial reproduction. However, the accumulation of high concentrations of ROS in cells can damage essential substances for life, including RNA, DNA, proteins, and lipids, thereby affecting bacterial survival rates (Guo et al., 2024). Ravichandiran et al. (2019) reported that treatment of SA with 1,4-naphthoquinone exerts antibacterial effects by promoting the production of ROS. Yan et al. (2024) indicated that the erythromycin polyketide compound TMC-154 can induce the production of ROS in *Streptococcus pyogenes*, thereby accelerating bacterial death (Yan et al., 2024). Hong et al. (2020) pointed out that ROS is a primary factor in the bactericidal action of quinolone drugs, and adjuvants that stimulate ROS production can enhance the lethality of quinolone drugs and other antimicrobial agents. In this study, we found that DIA can promote the release of intracellular ROS in MRSA. Electron microscopy observations showed that DIA can damage the cell membrane of MRSA, potentially exposing some redox enzymes to abnormal environments or interfering with their function, thereby triggering ROS production (Ribeiro et al., 2022). Transcriptome enrichment analysis indicated that DIA upregulated the glycolysis and tricarboxylic acid (TCA) cycle pathways in MRSA, leading to metabolic disorders and the accumulation of intermediate metabolites. The upregulation of glycolysis increased flux by enhancing the expression of key enzymes, meeting the cell’s ATP requirements (Zeden et al., 2023). The accumulation of intermediate metabolites (e.g., glyceraldehyde-3-phosphate) and the imbalance of NADH/NAD^+^ could generate ROS through non-enzymatic reactions (Zhong et al., 2024; Yu et al., 2024). The upregulation of the TCA cycle enhanced energy supply and carbon metabolism efficiency. The NADH and FADH₂ produced by the TCA cycle provided energy for bacterial growth, and its intermediate metabolites (e.g., *α*-ketoglutarate, oxaloacetate) were important precursors for the synthesis of macromolecules such as amino acids and nucleotides (Hou et al., 2023). The increased activity of the TCA cycle also helped maintain the cellular redox balance, enhanced the bacteria’s adaptability to environmental changes, and might improve the virulence and antibiotic resistance of certain pathogens. The accumulation of TCA cycle intermediate metabolites and electron leakage from the electron transport chain could further generate ROS (Ohkubo et al., 2022; Ohkubo et al., 2021). Moreover, as an external stimulus, DIA can activate its stress responses, which might initiate incomplete metabolic pathways or defense mechanisms. For instance, MRSA may produce oxidases to counteract the effects of DIA, but these oxidases can produce ROS during their functioning (Eld et al., 2022). Therefore, the increase in ROS is one of the factors leading to bacterial death, which may be associated with antibacterial activity of DIA.

A decrease in intracellular soluble protein content in bacterial cells can lead to multiple adverse effects, including impaired energy metabolism, difficulty in nutrient uptake and utilization, changes in cell morphology and reduced motility, and weakened environmental adaptability, thus severely impairing the survival, growth, and reproduction abilities of bacteria (Zhao et al., 2023). In our study, we found that DIA can significantly reduce the intracellular soluble protein content in MRSA, which may be due to DIA acting on ribosomes of MRSA, preventing mRNA from binding to ribosomes or interfering with the entry of tRNA, thus affecting protein synthesis and leading to a decrease in the total intracellular soluble protein content (Ni and Buszczak, 2023). Moreover, DIA may also interfere with the process of gene transcription into mRNA, such as by inhibiting the activity of RNA polymerase, preventing bacterial genes from being effectively transcribed into mRNA, and subsequently affecting subsequent translation steps, thereby reducing the synthesis of soluble proteins. The results of KEGG pathway enrichment from transcriptome sequencing indicate that DIA downregulates the ribosome metabolic pathway and the expression of genes involved in amino acid biosynthesis in MRSA. Meanwhile, it causes differential expression in purine, nucleotide, and pyrimidine metabolism. These changes collectively affect the content of soluble proteins within the cells. Specifically, when the ribosome metabolic pathway is downregulated, the synthesis and function of ribosomes are inhibited. This leads to a decrease in translation efficiency and protein production. Additionally, it triggers a stress response that impacts cell growth (Aleksandrova et al., 2024). The downregulation of the expression of genes related to amino acid biosynthesis restricts the supply of amino acids, thereby inhibiting the protein translation process. Moreover, the differential expression in purine, nucleotide, and pyrimidine metabolism affects the balance of nucleotides, reduces the efficiency of DNA replication, RNA transcription, and protein translation, and interferes with the energy supply and signal transduction of cells (Nolan et al., 2023). The combined effects of these metabolic pathways significantly reduce the content of soluble proteins in MRSA cells, which in turn weakens the metabolic capacity, stress response, and virulence of bacteria.

Increased fluidity of bacterial cell membranes can enhance drug uptake, making it easier for drugs to enter cells and exert antibacterial activity. It can also interfere with membrane protein function and promote the leakage of bacterial contents (Valliammai et al., 2021). This study demonstrates that DIA can promote the fluidity of MRSA cell membranes. As an external stimulus, DIA induces MRSA to activate its stress response mechanism. During this process, MRSA may synthesize certain substances with the function of regulating membrane fluidity, or alter the distribution of existing components on the cell membrane, thereby increasing its membrane fluidity.

Additionally, DIA exhibits antibiofilm activity against MRSA. When the concentration of DIA reaches 32 μg/mL, it significantly inhibits the formation of MRSA biofilms and shows a certain clearance capability against already formed biofilms. The study by Fu C et al. similarly reveals that DIA significantly inhibits the formation of *Enterococcus faecalis* biofilms at 16 μg/mL, and when the concentration is increased to 32 μg/mL or above, it effectively disperses pre-formed biofilms (Fu et al., 2023). Existing studies have shown that the production of ROS interferes with the formation of MRSA biofilms (Chen et al., 2022; Cáp et al., 2012). Through transcriptome sequencing and KEGG pathway enrichment analysis, it was discovered that differential gene expression in purine metabolism and nucleotide metabolism pathways affects the formation of MRSA biofilms. These findings collectively reveal the multi-faceted mechanisms by which DIA exerts its antibiofilm activity.

Despite extensive exploration of the antibacterial mechanisms of DIA, there are still some shortcomings that need to be addressed in future research. These are merely inferred based on transcriptome sequencing results and have not been tested in animal vivo environments. Therefore, subsequent studies should further validate its antibacterial activity through in animal vivo and cellular experiments, and use CRISPR gene editing to verify the specific target points against MRSA of DIA.

## Conclusion

In summary, DIA exhibits significant antibacterial activity against MRSA and is less likely to induce resistance, demonstrating synergistic antibacterial effects when used in combination with ampicillin. Additionally, DIA enhances the fluidity of the MRSA cell membrane and ROS release, reduces the synthesis of intracellular soluble proteins, inhibits the formation of MRSA biofilms and demonstrates good clearance effects on pre-formed biofilms. Transcriptome sequencing analysis revealed that DIA treatment of MRSA primarily affected multiple pathways, including microbial metabolism, ribosome pathway, tricarboxylic acid cycle, and nucleotide metabolism, thereby exerting antibacterial activity. Due to time and experimental constraints, we were unable to complete functional validation to further confirm the research findings. We fully acknowledge this limitation and will prioritize related experiments in subsequent studies to validate the functions of these genes, thereby refining the conclusions and enhancing the reliability of the results. In resistance induction experiments, DIA altered the colony morphology of MRSA, causing the disappearance of transparent hemolytic rings, but the specific mechanisms and reasons remain unclear and require further investigation. Overall, this study provides new ideas and strategies for the clinical application of DIA and also offers new theoretical and experimental foundations for exploring other non-antibacterial drug therapies for treating MRSA infections.

## Data Availability

The original contributions presented in the study are publicly available. This data can be found at: http://www.ncbi.nlm.nih.gov/bioproject/1227702/PRJNA1227702.

## Supplementary material

Supplementary Figure S1 Mass spectrometry identification diagrams of USA300 and its induced strains.

Supplementary Figure S2 Linear regression plot of normalized ROS over time. NC: Negative control; PC: Positive control; ns: no significant difference; ***, *P*< 0.001.
