# Supplementary material for: Exploring the antibacterial and anti-biofilm properties of Diacerein against methicillin-resistant Staphylococcus aureus
Source: Front Microbiol. 2025 Mar 20;16:1545902. doi: 10.3389/fmicb.2025.1545902 (PMC11965656; doi:10.3389/fmicb.2025.1545902)
Supplement: Supplementary file 1 [file Table_1.DOCX]

| **name** | **information** | **Base sequence (5’-3’)** | | **Tm** | **CG%** | **length** |
| --- | --- | --- | --- | --- | --- | --- |
| 16s | NZ_LN831048.1 | sense | AGCTCGTGTCGTGAGATGTTG | 58.4 | 52.4 | 194 |
|  |  | antisense | TCGCTGCCCTTTGTATTGTC | 58.7 | 50 |  |
| argF | AP017922 | sense | CGTGCTAAATATATTGGCACTGA | 58 | 39.1 | 228 |
|  |  | antisense | ACGGTATTCAATGCCATCATACA | 59.8 | 39.1 |  |
| deoD | AP017922 | sense | CAGTATTAATGCCTGGCGATC | 57.7 | 47.6 | 230 |
|  |  | antisense | ACAAGAACCGATACGAATGATTGT | 59.9 | 37.5 |  |
| lacA | AP017922 | sense | ATGGCGATTATTATTGGTTCAGAT | 59.4 | 33.3 | 197 |
|  |  | antisense | CCAAATGCATCGATAACAATACCT | 60.5 | 37.5 |  |
| pfkB | AP017922 | sense | TTGAACAGAGCAACAGCAACATA | 58.8 | 39.1 | 153 |
|  |  | antisense | TGCACTGTTATTTAATGTATCTGCA | 57.9 | 32 |  |
| purH | AP017922 | sense | GATTATCATGAAGTATTGACGCGAT | 59.9 | 36 | 203 |
|  |  | antisense | TGCTTAGCATTCGAAGTTCTCAC | 59.4 | 43.5 |  |
| purK | AP017922 | sense | CATTATTGGTGGTGGTCAGCTT | 59.3 | 45.5 | 106 |
|  |  | antisense | GTATCTACATGGACAATCTTCAGCA | 58.3 | 40 |  |

**Table S1 qPCR primer sequence list**
