# Supplementary figures and images for: Exploring the antibacterial and anti-biofilm properties of Diacerein against methicillin-resistant Staphylococcus aureus
Source: Front Microbiol. 2025 Mar 20;16:1545902. doi: 10.3389/fmicb.2025.1545902 (PMC11965656; doi:10.3389/fmicb.2025.1545902)

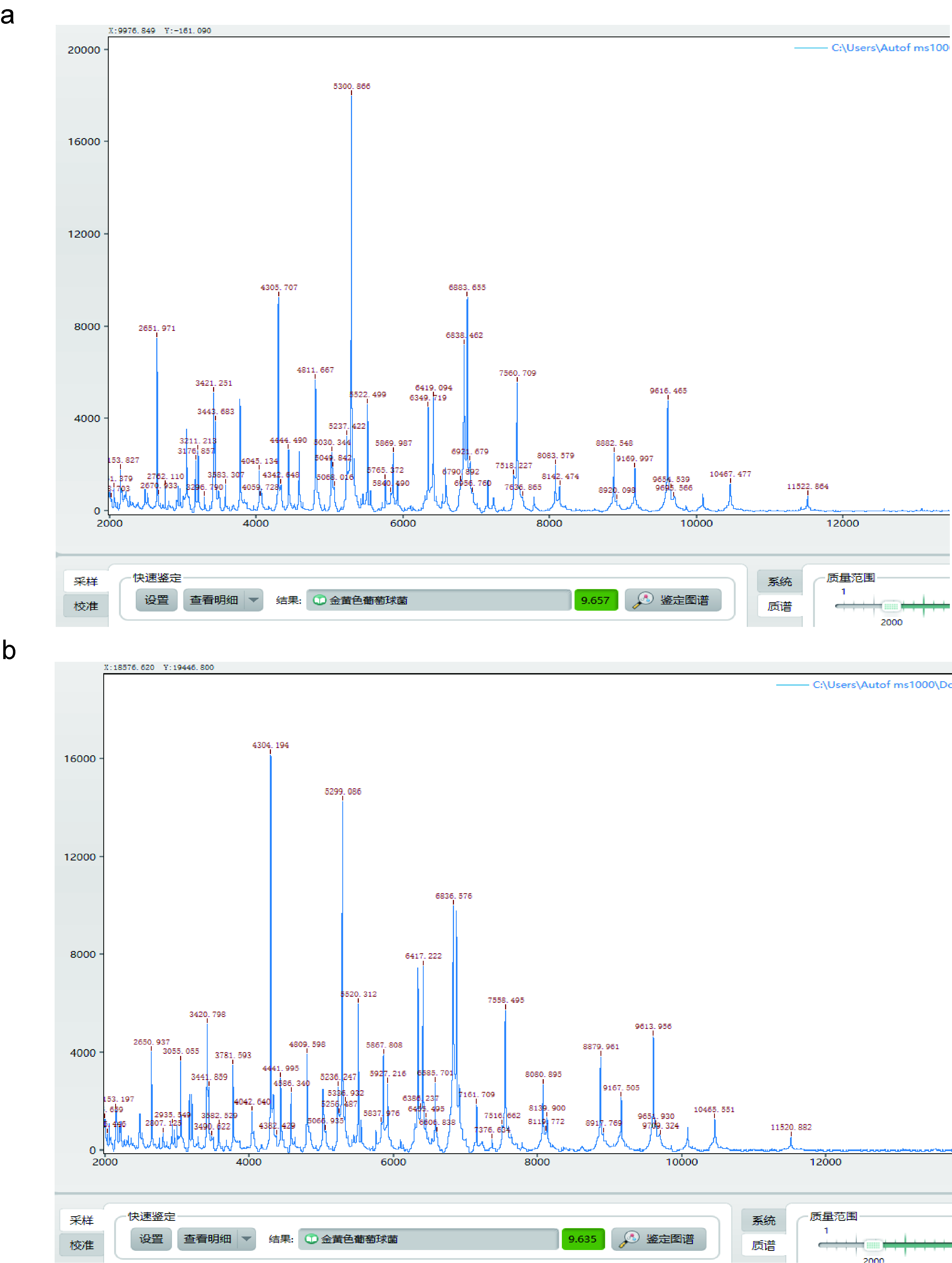

Supplement: Supplementary Figure S1 — Mass spectrometry identification diagrams of USA300 and its induced strains. [file Image_1.TIF]

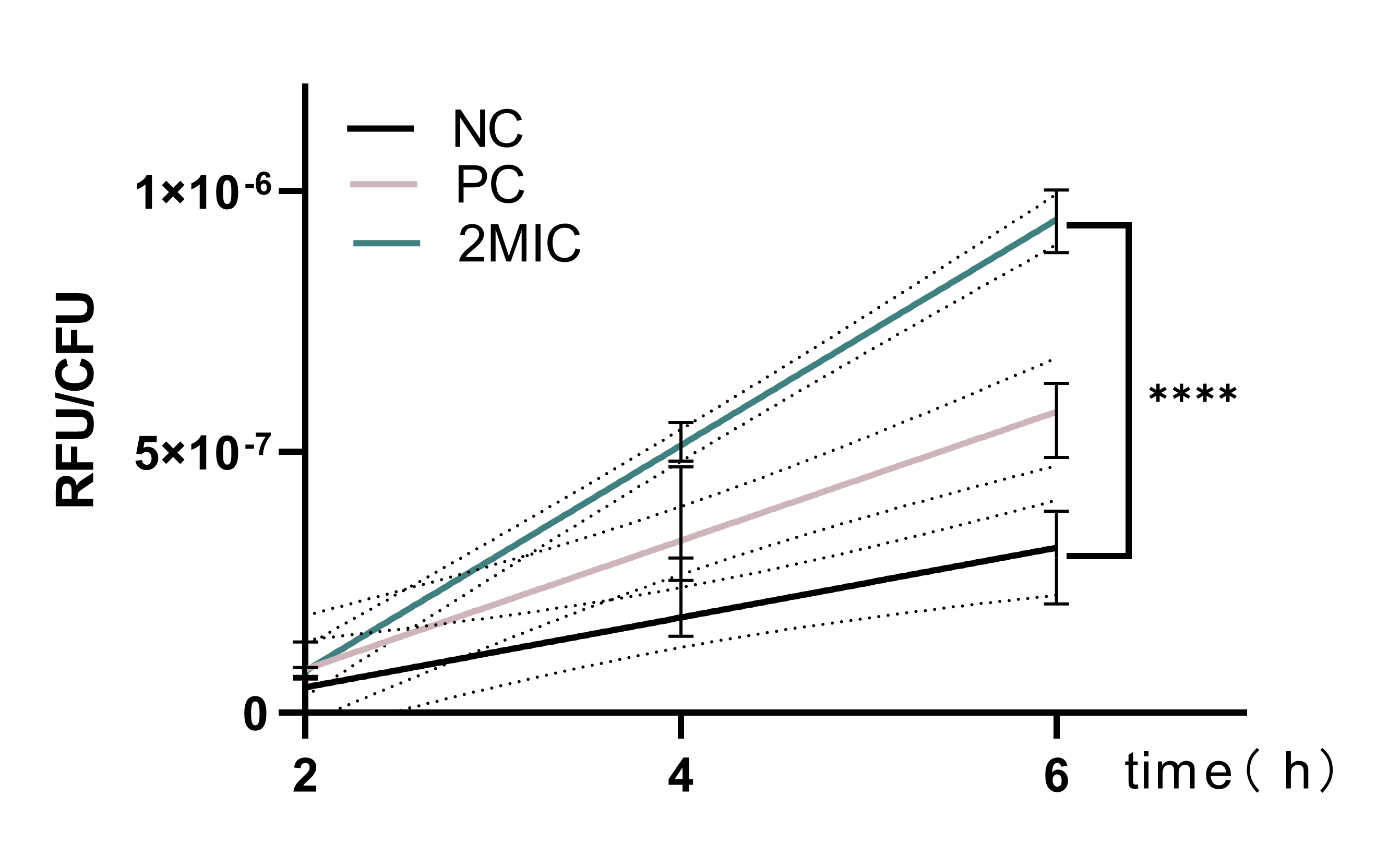

Supplement: Supplementary Figure S2 — Linear regression plot of normalized ROS over time. NC: Negative control; PC: Positive control; ns: no significant difference; ***, P< 0.001. [file Image_2.TIF]
